# Stochastic pharmacodynamics of a heterogeneous tumour-cell population

**DOI:** 10.1007/s10928-025-09974-7

**Published:** 2025-05-05

**Authors:** Van Thuy Truong, Paolo Vicini, James Yates, Vincent Dubois, Grant Lythe

**Affiliations:** 1 School of Mathematics, University of Leeds, Leeds, LS2 9JT UK; 2 Clinical Pharmacology and Quantitative Pharmacology, AstraZeneca, Granta Park, Cambridge, CB21 6GH UK; 3 Confo Therapeutics, Technologiepark 94, Zwijnaarde, 9052 Ghent, Belgium; 4 DMPK, Preclinical Sciences, RTech, GSK, Gunnels Wood Road, Stevenage, HRT SG1 2NY UK

**Keywords:** Targeted therapy, Heterogeneity, Cancer, Stochastic, Modelling, Pharmacodynamics

## Abstract

Standard pharmacodynamic models are ordinary differential equations without the features of stochasticity and heterogeneity. We develop and analyse a stochastic model of a heterogeneous tumour-cell population treated with a drug, where each cell has a different value of an attribute linked to survival. Once the drug reduces a cell’s value below a threshold, the cell is susceptible to death. The elimination of the last cell in the population is a natural endpoint that is not available in deterministic models. We find formulae for the probability density of this extinction time in a collection of tumour cells, each with a different regulator value, under the influence of a drug. There is a logarithmic relationship between tumour population size and mean time to extinction. We also analyse the population under repeated drug doses and subsequent recoveries. Stochastic cell death and division events (and the relevant mechanistic parameters) determine the ultimate fate of the cell population. We identify the critical division rate separating long-term tumour population growth from successful multiple-dose treatment.

**Supplementary Information:**

The online version contains supplementary material available at 10.1007/s10928-025-09974-7.

## Introduction

Models of the systemic distribution and elimination of drugs, and models of the effects of drugs, are called pharmacokinetic and pharmacodynamic (PK-PD) models [1–4]. Those based on ordinary differential equations (ODEs) are useful for ease of analysis [5–7] but they lack two important aspects of reality: stochasticity and heterogeneity. Heterogeneity is important in many contexts: protein expression varies from cell to cell; bacteria vary in their susceptibility to antibiotics [8]. Stochastic models have the feature that many outcomes are possible even if all conditions and initial states are given [9]; events occurring at random times impact disease progression and treatment effects. While agent-based models naturally incorporate heterogeneity and stochasticity and are amenable to computational studies [10], synthesis into simple formulae is rare [11, 12]. Here, we introduce and analyse stochastic models of a heterogeneous population of cancer cells acted on by a drug, which serve as a bridge between differential equation-based and agent-based models [9]. Following established practice in PK-PD modelling [13, 14], we consider both a single sustained dose and a regimen of multiple doses at regular intervals, with recovery periods between doses. In the first case, a constant drug concentration is maintained [15, 16]; in the second, the drug is absent during recovery periods.

We focus on a simple model of a drug acting to kill a population of tumour cells. The dynamics includes cell-to-cell variability: every tumour cell has a different value of an attribute that we call the regulator value and is linked to cell viability. Once the action of the drug succeeds in reducing a cell’s regulator value below a threshold, the cell is “in the death pool”, meaning it is subject to death. Our model is based on an agent-based model in which the regulator is phosphorylated ERK in vivo [12], but can also be applied to pharmacodynamics in vitro. Our motivating example was the reduction of phosphorylation of ERK by oral dose of the MEK inhibitor cobimetinib. The PD effect is modelled by a single variable that represents cellular pERK, as in the population-based PKPD model of Wong et al. [17] (used to fit tumour concentrations and pERK data) and the agent-based model with ODE approximations of Truong *et al* [12]. This PK-PD mechanistic framework is common to many oncology targeted treatments, and it serves as an illustrative example here. We are able to summarise the dynamics not by deriving ordinary differential equations valid when the number of cells is large, but in terms of survival functions and densities of extinction times with recognisable forms in the limit of large numbers of cells. The model is implemented in the python language and the code can be found online.^1^

Our model resembles the simplest pharmacodynamic models in that a cell’s regulator value decreases exponentially, with rate $\delta$, when the drug is present. The key stochastic aspect of the dynamics is the death of cells whose regulator value is sufficiently small. An advantage of a stochastic model is its natural endpoint: the death of the last tumour cell. The distribution of this extinction time can be constructed numerically and, in our simplest models, analytically. That is, we ask: how long until, under the action of the drug, all of *n* cells are eliminated? (Using ordinary differential equations it is possible to use the time a trajectory reaches a suitable lower bound as a proxy for the mean extinction time.)

**Fig. 1:**
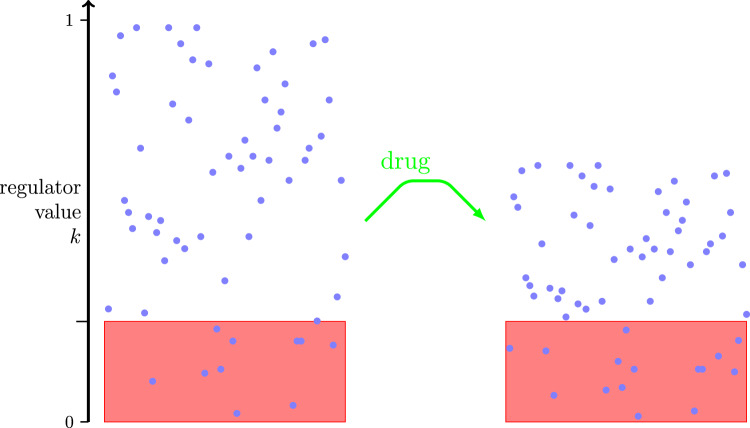
Each blue dot represents a tumour cell. The drug’s action reduces each cell’s regulator value, thereby increasing the fraction of cells in the death pool. Left: In a population of cells before drug dose, initial regulator values are uniformly distributed. Those cells with regulator values below a threshold (those inside the zone coloured red) are said to be in the death pool. Right: Some time after drug dose begins, each cell’s regulator value is reduced by the same factor. Hence, a greater proportion of cells are in the death pool (Color figure online)

A brief mathematical description of the assumptions and dynamics is as follows. The number of tumour cells at any time is an integer that may reach zero in finite time. Each cell has a regulator value, scaled to the interval (0, 1); we use values chosen from the uniform distribution on the interval as our initial condition. The action of the drug on an individual cell decreases its regulator value according to a deterministic relationship. In the schematic diagram, Fig. 1, each cell’s regulator value is multiplied by the factor 0.65 at some time after drug treatment begins (right panel). Examples from an agent-based model can be found in Figure 2.2 of the tutorial [12]. A cell’s death rate, in the stochastic sense where a rate is a probability per unit time, depends on its regulator value at time *t*, *k*(*t*), via the function *w*. That is, if a cell is alive at time *t* with regulator value *k*(*t*) then the probability that it dies before $t+\Delta t$ is $w(k(t))\Delta t$. If a cell is alive at time 0 with regulator value *k*, then the probability that it is still alive at time *t*, is found using the hazard-rate formula [18]

1 $$\begin{aligned} s(t,k) = {\mathbb {P}\left( \text {cell survives to time }t|k(0)=k\right) } = \exp \left( -\int _0^t w(k(s))\textrm{d}s\right) . \end{aligned}$$

Using (1), we calculate *S*(*t*), the probability that a tumour cell, chosen at random from the initial population, is still alive at time *t*. Two timescales are relevant in the analysis: $1/\delta$ describes the time for the drug to take effect and $1/\mu$ is the mean time for cells to die once they arrive in the death pool. Our multiple-dose-and-recovery scenario in Sect. 3 also contains a third timescale, $1/\lambda$, associated with division of tumour cells.

In Sect. 2, we assume the cancer cell population is continuously exposed to the drug. In the first subsection, we examine the fate of a single cell: Once its regulator value *k* falls below a certain threshold due to drug exposure, it enters the death pool. (The threshold value of 0.25 is chosen to match the corresponding published agent-based model in Truong *et al* [12] but can be adjusted to the pathway of interest.) While death is the ultimate fate of any cell, the mean time to extinction depends on its initial *k* value. We investigate the survival probability when *w*(*k*) is constant below the threshold and, in the Supplementary Material, with *w*(*k*) a linear function of *k* below the threshold. (Which is more realistic will depend on the physiological pathway of interest.) The survival probability is given in terms of the drug-specific parameters $\mu$ and $\delta$. In the second subsection, we look into a population where each cell has a different *k* value. For a population with *n* individual cells we derive the expectation and variance of the time to population extinction. With increasing *n*, the probability density of extinction times shifts towards later times while maintaining its shape. 

In Sect. 3, cell division is included in the dynamics by means of a second threshold regulator value. The range of regulator values, and hence the population of cells at any time, is divided into three: cells with values over the threshold of 0.5 are said to be the division pool because they have a constant probability per unit time of dividing into two cells; cells with k in the range (0.25, 0.5) neither divide nor die; cells with values below 0.25 are said to be in the death pool (Similarly to Sect. (), the threshold value of 0.5, in principle arbitrary, is chosen to match the corresponding published agent-based model in Truong *et al* [12]. In Sect. 2, we consider a continuous drug dose that drives all cells into the death pool, hence no division occurs.)

Although our calculations focus on nondimensional combinations of parameters, such as $\delta /\mu$, it is helpful to keep in mind reasonable timescales that depend on context. We expect $1/\delta$, the time required for the drug to alter the biochemical state of cells, in vitro or in vivo, to be minutes or hours. Mean times for the processes of cell death and cell division, $1/\mu$ and $1/\lambda$, will be hours or days [17, 19]. Yang et al. [19] used time-resolved microscopy to count number of live and dead tumour cells over four days in vitro. They estimated a division rate of 0.1 day$^{-1}$.

Also in Sect. 3, we consider multiple rounds of drug dose and recovery [20], with cell death and division. Drug treatment is repetitively administered with a recovery period before the next cycle. During recovery periods, the *k* value of each cell increases; some cells enter the division pool and divide. Thus we find dynamics where the number of cells tends to decrease during drug doses and to increase during recovery intervals. Under the influence of repeated doses and recovery periods, whether extinction is the ultimate fate of the population depends on the balance between cell death and division. That balance, in turn, depends on the distribution of regulator values in the surviving cell population, which becomes biased towards larger values. We derive equations to describe the change of the regulatory value *k* after multiple cycles. Thus, the calculations begin with a single cell with known initial regulator value, then consider a randomly-selected cell from the distribution of initial regulator values, allowing consideration of a cohort of *n* cells under single sustained dose. The model then extends to multiple rounds of dose and recovery, including cell division. An overview is provided in Table 1. Additionally, in the Supplementary Material, the relationship between the critical death rate $\mu _c$ needed to be attained by the drug, the cellular division rate $\lambda$, the drug potency $\delta$, and schedule-specific parameters cycle time *T* and recovery time $T_d$, is investigated.

Complicated dynamical systems are most useful as models of real phenomena when the parameters can be combined into formulae that summarise the behaviour. In models with stochasticity and heterogeneity, such a summary must go beyond mean quantities by predicting distributions of outcomes. We are able to provide such formulae and distributions, with particular focus on the distribution of times until the the elimination of the last cancer cell.

**Table 1:** Overview of model framework and results

Single cell behaviour (known *k* value)	Randomly-selected cell	Population of *n* cells under single sustained dose	Multiple-dose treatment, including cell division
Time of arrival in the death pool $t_k$	Survival probability *S*(*t*) with a step-function death rate	Mean and variance of time to population extinction $\tau _n$	Dynamics of regulator values under multiple-dose treatment using a difference equation
Survival probability *s*(*t*, *k*)	Mean and variance of time to death of a single cell $\tau _1$	Probability density $f_n(t)$ of the time to population extinction	Estimate of the critical death rate $\mu _c$ for population extinction

## Single sustained dose, no cell division

**Fig. 2:**
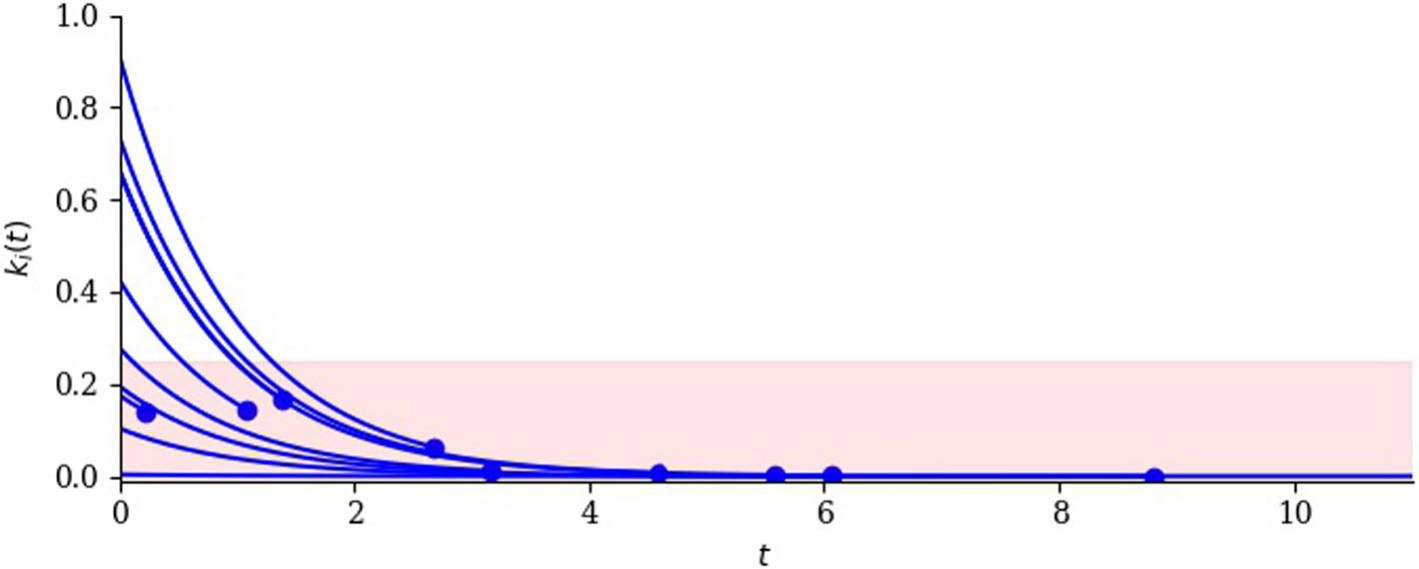
Illustrating the effect of a sustained dose on a population of 10 cells. Each blue line is the regulator attribute of one cell as a function of time. Lines terminate in blue dots that indicate cell death. Cells are said to be in the death pool when their regulator value is less than 0.25, indicated by red shading. Simple model without cell division, with parameter values $\delta =0.2$ and $\mu =1$

We assume that an uninterrupted drug dose (giving rise to constant drug levels) yields the following deterministic relationship for cell *i*

2 $$\begin{aligned} k_i(t)=k_i(0)\exp (-\delta t), \end{aligned}$$

and that cells with $k_i(t)<0.25$ are in the death pool. In the simplest case, the death rate of any cell in the death pool is equal to a constant $\mu$. Cells with $k_i(0)>0.25$ are not initially in the death pool but enter the pool when their regulator value has decreased to 0.25. Figure 2 is a small-scale illustration of the resulting dynamics: the ten cells initially present die, one by one, as their regulator values decline under the influence of the drug, taking them into the death pool.

Let $t_k$ be the first time that a cell, whose initial regulator value is equal to *k*, enters the death pool. The lifetime of the cell is the sum of $t_k$ and the time to die once the cell enters the death pool. We can calculate $t_k$ using (2):

3 $$\begin{aligned} t_k = \inf \{t\ge 0:\ k_i(t) \le 0.25\ |\ k_i(0)=k\} \ = \ {\left\{ \begin{array}{ll} 0 & \qquad 0\le k \le \frac{1}{4}\\ \dfrac{1}{\delta }\log (4k) & \qquad \frac{1}{4} < k \le 1. \end{array}\right. } \end{aligned}$$

**Fig. 3:**
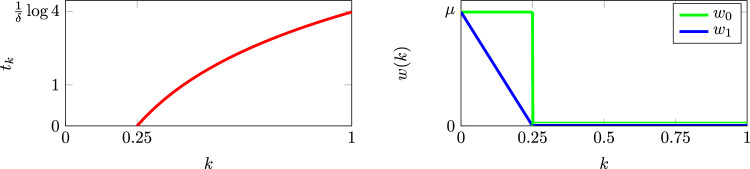
Left: The time, $t_k$, at which a cell enters the death pool is shown as a function of the cell’s initial scaled regulator value. Right: two death-rate functions $w_0(k)$ and $w_1(k)$

This time is shown on the LHS in Fig. 3. On the RHS, two possible functions *w*(*k*) are shown. We will use $w_0(k)$ in this Section and $w_1(k)$ in the Supplementary Material, where

4 $$\begin{aligned} w_0(k)={\left\{ \begin{array}{ll} 0 & k>\frac{1}{4}\\ \mu & k<\frac{1}{4}.\end{array}\right. } \end{aligned}$$

### Survival and death of individual cells

Consider a cell chosen at random from a population with initial regulator values uniformly distributed between 0 and 1. The probability that it survives to *t* is obtained by integrating (1):

5 $$\begin{aligned} S(t) = {\mathbb {P}\left( \text {randomly-chosen cell survives to time }t\right) } = \int _0^1 s(t,k) \textrm{d}k. \end{aligned}$$

We evaluate the integral in (1) using (4) to yield the probability that a cell labelled *i* survives to time *t*:

6 $$\begin{aligned} s(t,k) = {\mathbb {P}\left( \text {cell survives to time }t|k_i(0)=k\right) }= {\left\{ \begin{array}{ll} 1 & \qquad t \le t_k\\ \textrm{e}^{-\mu (t-t_k)}& \qquad t>t_k. \end{array}\right. } \end{aligned}$$

**Fig. 4:**
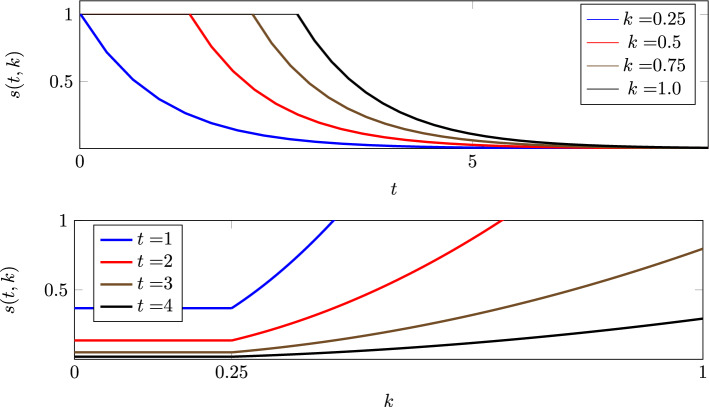
Upper: *s*(*t*, *k*) is the probability that a cell, whose initial regulator value is *k*, is still alive at time *t* when $w(k)=w_0(k)$. If *k* is fixed then *s*(*t*, *k*) is a non-increasing function of *t*. Lower: The probability that a cell, whose initial regulator value is *k*, is still alive. If *t* is fixed then *s*(*t*, *k*) is a non decreasing function of *k*. The formula used is (6), with $\mu =1$ and $\delta =0.5$

Figure 4 shows *s*(*t*, *k*) as a function of *t* using four values of *k* (upper) and as a function of *k* using four values of *t* (lower).

Next, consider a cell chosen at random from a population with initial regulator values uniformly distributed between 0 and 1. The probability that a cell, chosen in this way, survives to *t* is found by evaluating the integral in (5) as follows:

7 $$\begin{aligned} S(t)&= \frac{1}{4}\textrm{e}^{-\mu t} +\int _{\frac{1}{4}}^1s(t,k)\textrm{d}k\end{aligned}$$

8 $$\begin{aligned}&= \frac{1}{4}\textrm{e}^{-\mu t} + {\left\{ \begin{array}{ll}\textrm{e}^{-\mu t} \displaystyle \int _{\frac{1}{4}}^{\frac{1}{4}\textrm{e}^{\delta t}} (4k)^{\mu /\delta }\textrm{d}k + 1-\frac{1}{4}\textrm{e}^{\delta t}& \hspace{28.45274pt}\delta t<\log 4\\ \textrm{e}^{-\mu t} \displaystyle \int _{\frac{1}{4}}^1 (4k)^{\delta /\mu } \textrm{d}k& \hspace{28.45274pt}\delta t \ge \log 4. \end{array}\right. } \end{aligned}$$

Note that, if $k>\frac{1}{4}$ then $\textrm{e}^{\mu t_k} = (4k)^{\mu /\delta }$. On the RHS in (8), the term $\frac{1}{4}\textrm{e}^{-\mu t}$ corresponds to cells that are in the death pool at $t=0$ (and remain there). The term $1-\frac{1}{4}\textrm{e}^{\delta t}$ is the fraction of cells that are not in the death pool. Evaluating the integrals,

9 $$\begin{aligned} S(t) = {\left\{ \begin{array}{ll}1- \dfrac{\mu }{4}\dfrac{\textrm{e}^{\delta t}-\textrm{e}^{-\mu t}}{\delta +\mu } & \qquad \delta t \le \log 4\\ A\textrm{e}^{-\mu t}\qquad & \qquad \delta t \ge \log 4, \end{array}\right. } \end{aligned}$$

where

10 $$\begin{aligned} A = \int _0^1\textrm{e}^{\mu t_k}\textrm{d}k = \dfrac{4^{\mu /\delta }+\frac{1}{4}\frac{\mu }{\delta }}{1+\frac{\mu }{\delta }}. \end{aligned}$$

The factor *A* is an increasing function of the ratio $\mu /\delta$, with $A=1$ when $\mu /\delta =0$. In the limit $\mu /\delta \rightarrow 0$, the action of the drug is fast compared to the typical survival time of a cell in the death pool.

The probability density of single-cell death times, shown as the top panel in Fig. 6, is obtained from the derivative of *S*(*t*):

11 $$\begin{aligned} f(t) = -S'(t) = {\left\{ \begin{array}{ll} \dfrac{\mu \delta \textrm{e}^{\delta t}-\mu ^2\textrm{e}^{-\mu t}}{4(\delta +\mu )} & \qquad \delta t \le \log 4\\ \mu A\textrm{e}^{-\mu t}\qquad & \qquad \delta t \ge \log 4. \end{array}\right. } \end{aligned}$$

The mean time to death of a single cell will be denoted $\textrm{I}\!\textrm{E}(\tau _1)$. It is equal to the sum of the mean time to arrive in the death pool plus the mean time to die once in the death pool:

12 $$\begin{aligned} \textrm{I}\!\textrm{E}(\tau _1) = \int _{\frac{1}{4}}^1t_k\textrm{d}k + \frac{1}{\mu } = \frac{1}{\delta }(\log 4-\frac{3}{4})+ \frac{1}{\mu }. \end{aligned}$$

The variance of $\tau _1$ is

13 $$\begin{aligned} \text {var}(\tau _1) = \dfrac{1}{\mu ^2}. \end{aligned}$$

Note that the variability of the mean time to death of a single cell is a function only of the time the cells take to die once they arrive in the death pool.

### Extinction of a cohort of *n* tumour cells

**Fig. 5:**
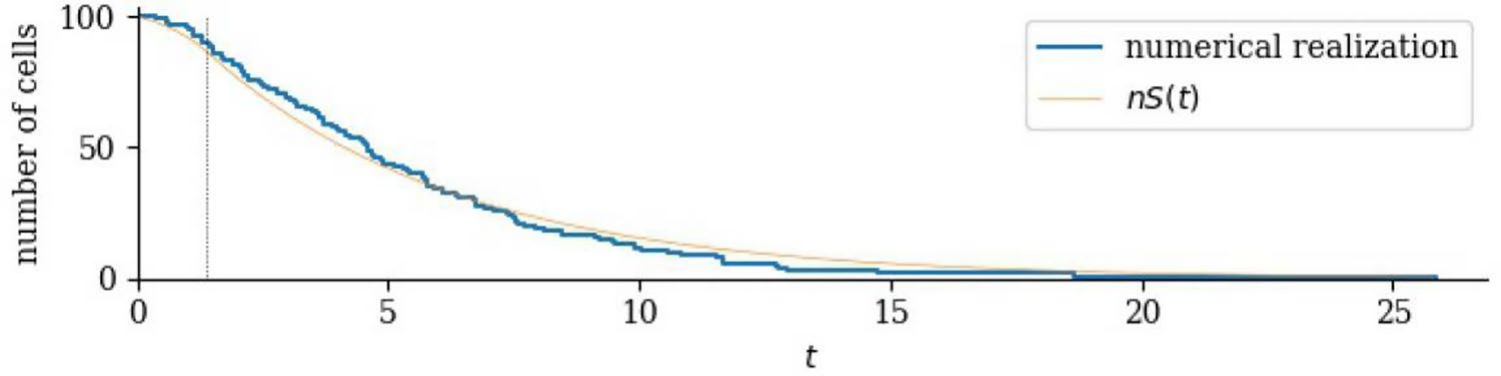
Blue: The number of surviving cells as a function of time in one realisation of the single, sustained dose model. Also shown is the smooth function obtained by averaging over many realisations, equal to the survival function *S*(*t*) (9) multiplied by the initial number of cells. The vertical dotted line indicates $t=\log 4/\delta$. Here $n=100$, $\delta =1$ and $\mu =0.2$ (Color figure online)

Suppose there are *n* tumour cells at $t=0$, with regulator values uniformly distributed in (0, 1). How long until all *n* cells die? An example realisation, with $n = 100$, calculated using the single sustained dose model described above, is shown in Fig. 5. That is, the blue line is a number of cells surviving to time *t* when each, independently, is assigned an initial regulator value in (0, 1) and, under the action of the drug, enters the death pool.

We define the random variable $N_t$ to be the number of cells alive at time *t*, with $N_0=n$. Let $\tau _n$ be the first time that $N_t=0$. Inspecting (9), we see that the single-cell survival probability has a simple exponential form as long as $\delta t > \log 4$. The form is $S(t)=A\textrm{e}^{-\mu t}$ is found if an individual lifetime is drawn as a random variable that is the sum of a fixed time of duration $\log A/\mu$ and an exponentially-distributed time with mean $1/\mu$. The time to extinction of *n* such individuals ($\textrm{I}\!\textrm{E}(\tau _n)$) is given by [21]

14 $$\begin{aligned} \textrm{I}\!\textrm{E}(\tau _n) = \frac{1}{\mu }\left( \log A +1 + \frac{1}{2}+\frac{1}{3}+\cdots +\frac{1}{n}\right) \simeq \frac{1}{\mu }\left( \log nA + \gamma \right) , \end{aligned}$$

where $\gamma =0.577\ldots$. We use the symbol $\simeq$ to denote the large-*n* approximation. Similarly [22, 23]

15 $$\begin{aligned} \text {var}(\tau _n) = \frac{1}{\mu ^2}\left( 1+\frac{1}{4}+\frac{1}{9}+\cdots +\frac{1}{n^2}\right) \simeq \frac{1}{\mu ^2}\frac{\pi ^2}{6}. \end{aligned}$$

**Fig. 6:**
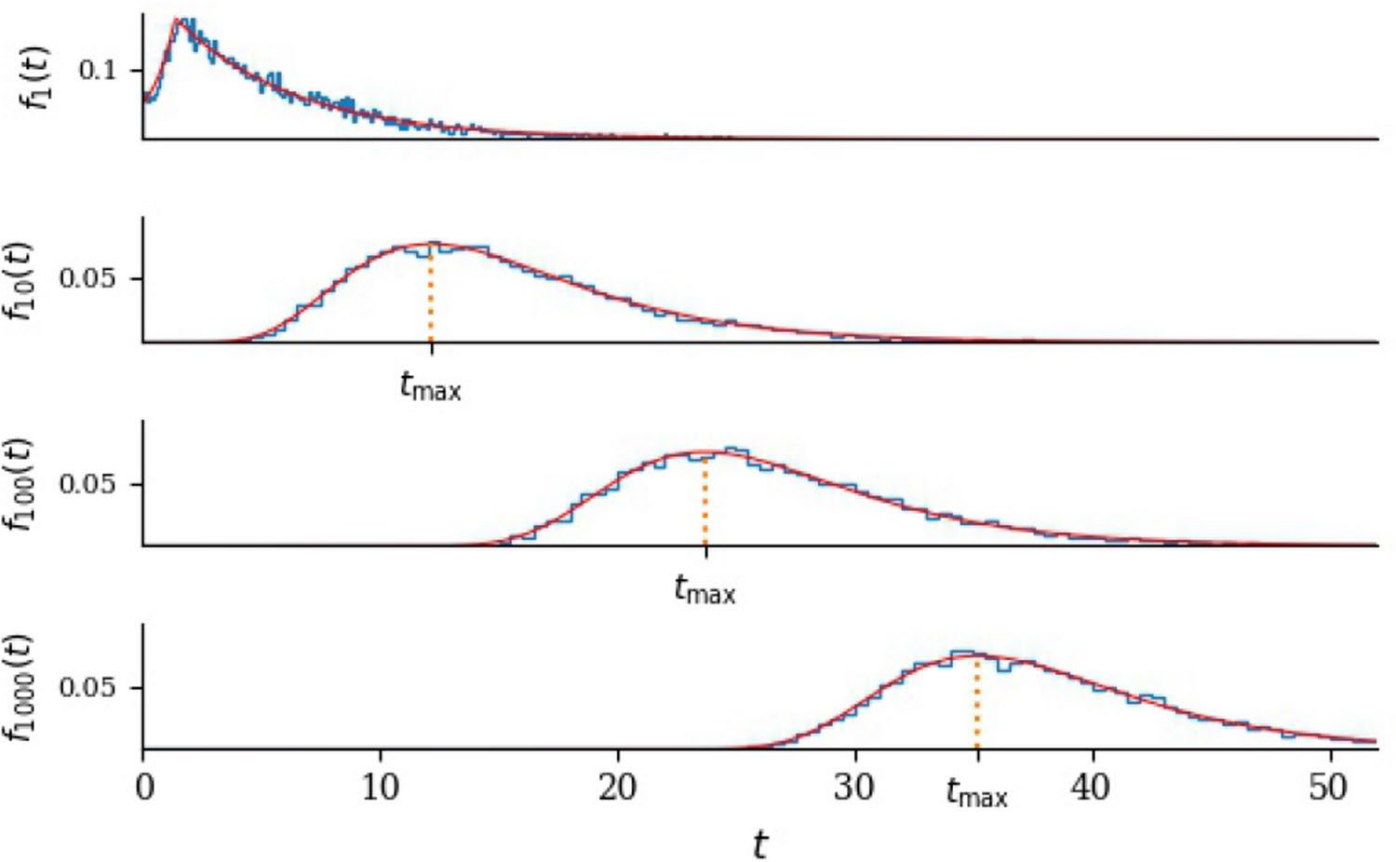
Probability density of extinction times, sustained dose with $n=1$, 10, 100 and 1000. Solid red lines are the exact formulae; the blue histograms are compiled from 10,000 numerical realisations. The same horizontal scale is used in each case, with $\mu =0.2$ and $\delta =1$. Top: $n=1$. The maximum is at $t=\frac{1}{\delta }\log 4$, after which all cells are in the death pool. In each of the lower three panels, the vertical dotted line is $t_\textrm{max}=\frac{1}{\mu }\log (nA)$. The ratio $\mu /\delta$ determines the factor *A*

As can be seen in Figs. 5 and 6, a considerable simplification arises because typical values of $\tau _n$ are large compared to $1/\delta$. When $\delta t>\log 4$, ${\mathbb {P}\left( \text {randomly-chosen cell dies before time }t\right) }=1-A\textrm{e}^{-\mu t}$, and we are able to derive the probability density of $\tau _n$ explicitly. Because each cell is independent, when $\delta t>\log 4$,

16 $$\begin{aligned} {\mathbb {P}\left( \tau _n<t\right) } = {\mathbb {P}\left( n \text { cells all die before }t\right) } = (1-A\textrm{e}^{-\mu t})^n, \end{aligned}$$

and the probability density of $\tau _n$ is

17 $$\begin{aligned} f_n(t) = \mu nA\textrm{e}^{-\mu t} \left( 1-A\textrm{e}^{-\mu t}\right) ^{n-1}, \end{aligned}$$

which attains its maximum value when $nA\textrm{e}^{-\mu t}=1$. Figure 6 shows the density with different choices of *n*. The maximum of the density is at $t_\textrm{max}= \frac{1}{\mu }\log {nA}$. It is striking, in Fig. 6, that increasing *n* while keeping $\mu$ constant shifts the distribution to the right, maintaining its shape. With this in mind, consider (16) when *t* is close to $t_\textrm{max}$ and *n* is large so that $A\textrm{e}^{-\mu t}$ is small. Then $\log (1-A\textrm{e}^{-\mu t})^n = n\log (1-A\textrm{e}^{-\mu t}) \simeq -nA\textrm{e}^{-\mu t}$, so that ${\mathbb {P}\left( \tau _n<t\right) } = \exp (-nA\textrm{e}^{-\mu t})$. If $T_n = \mu t-\log (nA)$ then ${\mathbb {P}\left( \tau _n<t\right) }\simeq \exp (-\textrm{e}^{-T_n})$ and

18 $$\begin{aligned} f_n(t) \simeq \mu \exp \left( -T_n-\textrm{e}^{-T_n} \right) . \end{aligned}$$

In other words, the random variable $\mu (\tau _n-t_\textrm{max})$ is approximately Gumbel-distributed [24] when *n* is large.

We use (18) to construct an algorithm that directly generates samples from the extinction-time density without simulating the whole timecourse of the stochastic process. Given any *p* in (0, 1), the value of *t* such that ${\mathbb {P}\left( \tau _n<t\right) }=p$ is^2^

19 $$\begin{aligned} t&= t_\textrm{max}-\frac{1}{\mu }\log (\log (\frac{1}{p})). \end{aligned}$$

**Fig. 7:**
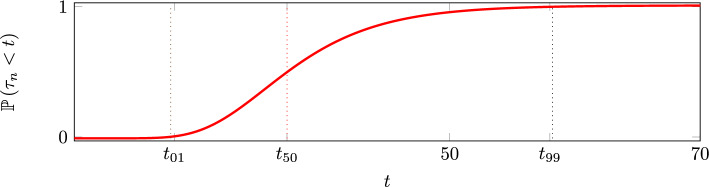
The cumulative density function of the extinction time of a cohort of $n=1000$ cells. The times satisfying ${\mathbb {P}\left( \tau _n<t\right) }=0.01$, ${\mathbb {P}\left( \tau _n<t\right) }=0.50$ and ${\mathbb {P}\left( \tau _n<t\right) }=0.99$ are shown as dotted vertical lines marked $t_{01}$, $t_{50}$ and $t_{99}$. The values are $t_{01} = t_\textrm{max}-1.50/\mu$, $t_{50} = t_\textrm{max}+0.37/\mu$ and $t_{99} = t_\textrm{max}+4.6/\mu$, where $t_\textrm{max}=\frac{1}{\mu }\log nA$. The curve is plotted using $\mu =0.2$ and $\delta =1$, so that $A=1.14$

Thus if $\textbf{U}$ is uniformly distributed in (0, 1) (the simplest random variable available in modern computer languages [10]) then the random variable $\left( -\log (-\log \textbf{U}) + \log (nA)\right) /\mu$ is a sample from the density (18). In Fig. 7, we display the cumulative distribution of the extinction time $\tau _{1000}$. The Figure also indicates $t_{01}$, $t_{50}$ and $t_{99}$, defined as the values of *t* such that ${\mathbb {P}\left( \tau _n<t\right) }$ is equal to 0.01, 0.50 and 0.99. The factor *A* is calculated using the same parameter values as in Fig. 6.

In Fig. 7 we display the cumulative distribution of the extinction time of a population consisting of 1000 cells. The Figure also indicates $t_{01}$, $t_{50}$ and $t_{99}$, defined as the values of *t* such that the probability of population extinction ${\mathbb {P}\left( \tau _n<t\right) }$ is equal to 0.01, 0.50 and 0.99, calculated using (19). The constant *A* is calculated using the same parameter values as in Fig. 6. In Table 2, we summarise the main results of the sustained-dose model.

**Table 2:** Main formulae associated with the sustained single dose model

	Formula	Number
Single-cell time to reach the death pool $t_k$	$\dfrac{1}{\delta }\log (4k)$	(3)
Single-cell survival	$\textrm{e}^{-\mu (t-t_k)}$	(6)
Fraction of single cells surviving	$A\textrm{e}^{-\mu t}$	(9),(10)
Single-cell mean time to extinction	$\dfrac{1}{\delta }(\log 4-\frac{3}{4})+ \dfrac{1}{\mu }$	(12)
Single-cell variance time to extinction	$\dfrac{1}{\mu ^2}$	(13)
Population mean extinction time	$\dfrac{1}{\mu }\left( \log nA + \gamma \right)$	(14)
Population variance extinction time	$\dfrac{1}{\mu ^2}\dfrac{\pi ^2}{6}$	(15)

## Multiple-dose treatment with cell division

In multiple-dose treatment, the drug is administered in doses of duration $T_d$ each, followed by a recovery period of duration $T-T_d$. Thus, one cycle takes time *T*. In the example of Fig. 9, the recovery period is twice the dose duration.

**Fig. 8:**
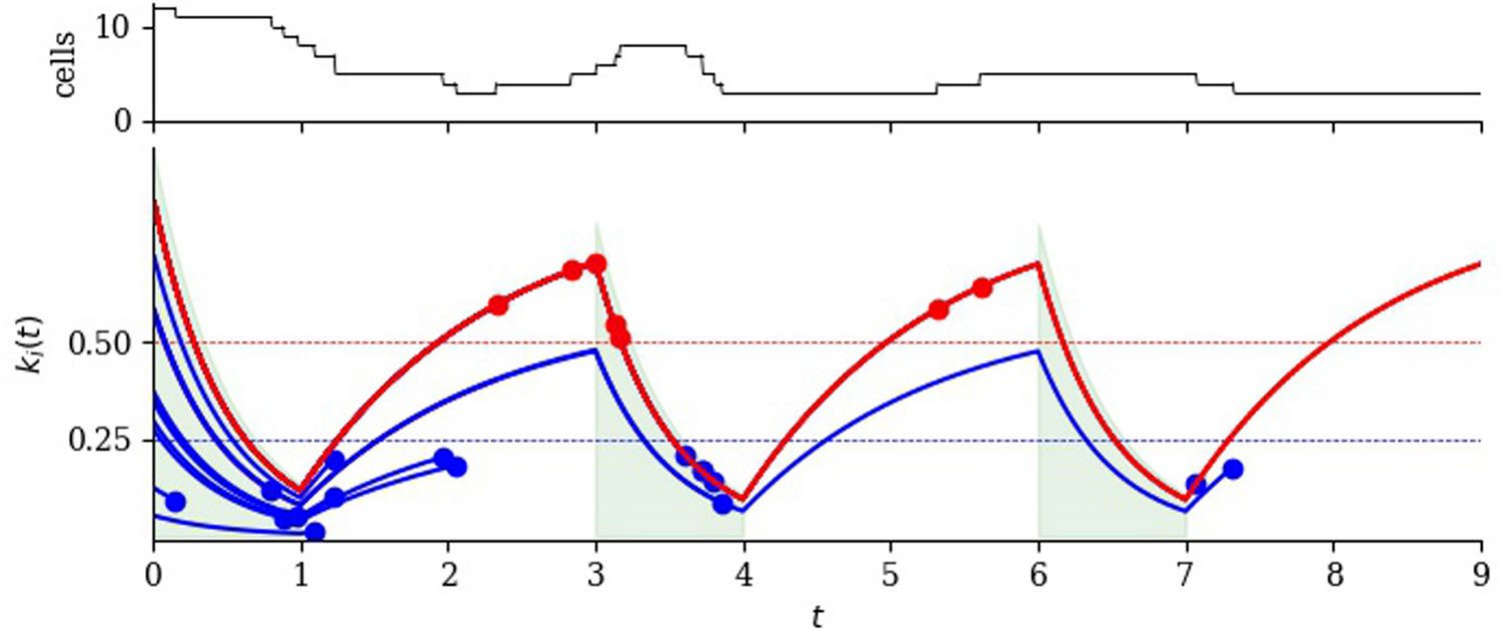
Illustrating the effect of multiple cycles of drug dose and recovery on a small cell population. The number of cells as a function of time is shown in the upper panel. Doses have duration one time unit, starting at $t=0$, $t=3$ and $t=6$ (green shading in the lower panel). Each recovery duration is two time units. Blue lines represent cells that die before the end of the third cycle and red lines represent cells that survive to the end of the third cycle. Blue dots indicate the death of a cell, which happens with rate $\mu$ to cells with regulator values smaller than 0.25 (below the blue dashed line). Red dots indicate cell division, which happens with rate $\lambda$ to cells with regulator values greater than 0.5 (above the red dashed line). In the initial cell population, regulator values are uniformly distributed between 0 and 1. Note that, after three rounds of dose and recovery, all remaining cells are descended from the initial cell with the highest initial regulator value. The parameter values are $\mu =1$, $\lambda =0.4$, $\delta =2.5$, $\alpha =2$, $T=3$ and $T_d=1$ (Color figure online)

Let us examine how each cell’s regulator value changes. While a dose is being administered, the drug’s effect is similar to that described by (2):

$$\begin{aligned} k_i(t) = k_i(0)\textrm{e}^{-\delta t},\qquad \qquad 0 \le t \le T_d. \end{aligned}$$

In recovery periods, on the other hand, a cell’s regulator value relaxes towards its natural value $k_i(0)$ with rate $\alpha$. In the first recovery period

$$\begin{aligned} k_i(t) = k_i(0) - (k_i(0)-k_i(T_d))\textrm{e}^{-\alpha (t-T_d)},\qquad \qquad T_d<t<T. \end{aligned}$$

In general, during the *n*th dose,

$$\begin{aligned} k_i(t) = k_i(nT)\textrm{e}^{-\delta (t-nT)}, \qquad \qquad nT< t < nT + T_d. \end{aligned}$$

In the *n*th recovery period

$$\begin{aligned} k_i(t) = k_i(0) - (k_i(0)-k_i(nT+T_d))\textrm{e}^{-\alpha (t-nT-T_d)},\qquad \qquad nT+T_d< t < (n+1)T. \end{aligned}$$

**Fig. 9:**
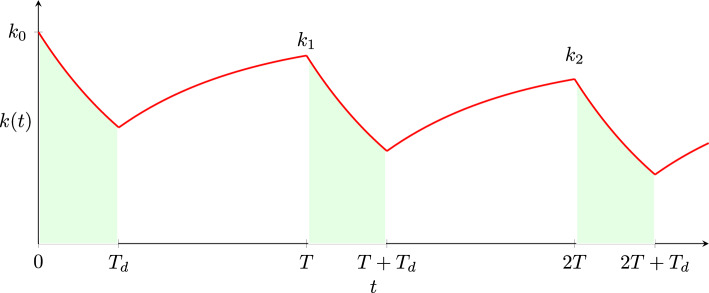
The regulator value of an individual cell under multiple-dose treatment is shown in red. Drug doses are administered for time $T_d$ (indicated by green shading) followed by a recovery period. The total cycle time is *T*. The regulator value at the *n*th peak value is denoted $k_n$ and given by (21)

As shown in Fig. 9, we denote the *n*th peak regulator value by $k_n$. That is, $k_n = k_i(nT)$. Then

$$\begin{aligned} k_1 = k_0(1-\textrm{e}^{-\alpha (T-T_d)}) + k_0\textrm{e}^{-\delta T_d}\textrm{e}^{-\alpha (T-T_d)}. \end{aligned}$$

Given $n\ge 1$, we find that $k_{n+1}$ depends on $k_n$ and $k_0$ as follows:

20 $$\begin{aligned} k_{n+1} = ak_0 + bk_n, \end{aligned}$$

where $a=1-\textrm{e}^{-\alpha (T-T_d)}$ and $b=\textrm{e}^{-\delta T_d}\textrm{e}^{-\alpha (T-T_d)}$. The solution of (20) is

21 $$\begin{aligned} k_n = K + (k_0-K)b^n\qquad \text { where }K = \frac{a}{1-b}k_0. \end{aligned}$$

As $n\rightarrow \infty$, $k_n\rightarrow K$. We observe that the asymptotic peak value, *K*, is a function of *T* and $T_d$, $\delta$ and $\alpha$; that is, it depends on the dosing duration and effectiveness, and on the extent of recovery after each dose. The parameters, interpretation and reasonable values are summarised in Table 3.

**Table 3:** Parameters of the multiple-dose model, with cell death and division

Symbol	Dimensions	Interpretation	Simulation value	Published parameter ranges or potential experimental design
$\mu$	T$^{-1}$	Cell death	1, 0.2	Measurable by exposing tumour cell populations to different constant drug concentrations
$\lambda$	T$^{-1}$	Cell division	0.4, 0.25	0.1 day$^{-1}$ [19], 0.0828 day$^{-1}$ [17]
$\delta$	T$^{-1}$	Drug action	0.2, 1, 2.5	0.713 day$^{-1}$ for 0.8 mg/kg,0.279 day$^{-1}$ for 0.2 mg/kg,0.159 day$^{-1}$ for 0.05 mg/kg(fitted from [17])
$\alpha$	T$^{-1}$	Relaxation	2	0.074 day$^{-1}$ for 0.8 mg/kg,0.012 day$^{-1}$ for 0.2 mg/kg,0.036 day$^{-1}$ for 0.05 mg/kg(fitted from [17])
*T*	T	Cycle time	3	
$T_d$	T	Dose time	1	

**Fig. 10:**
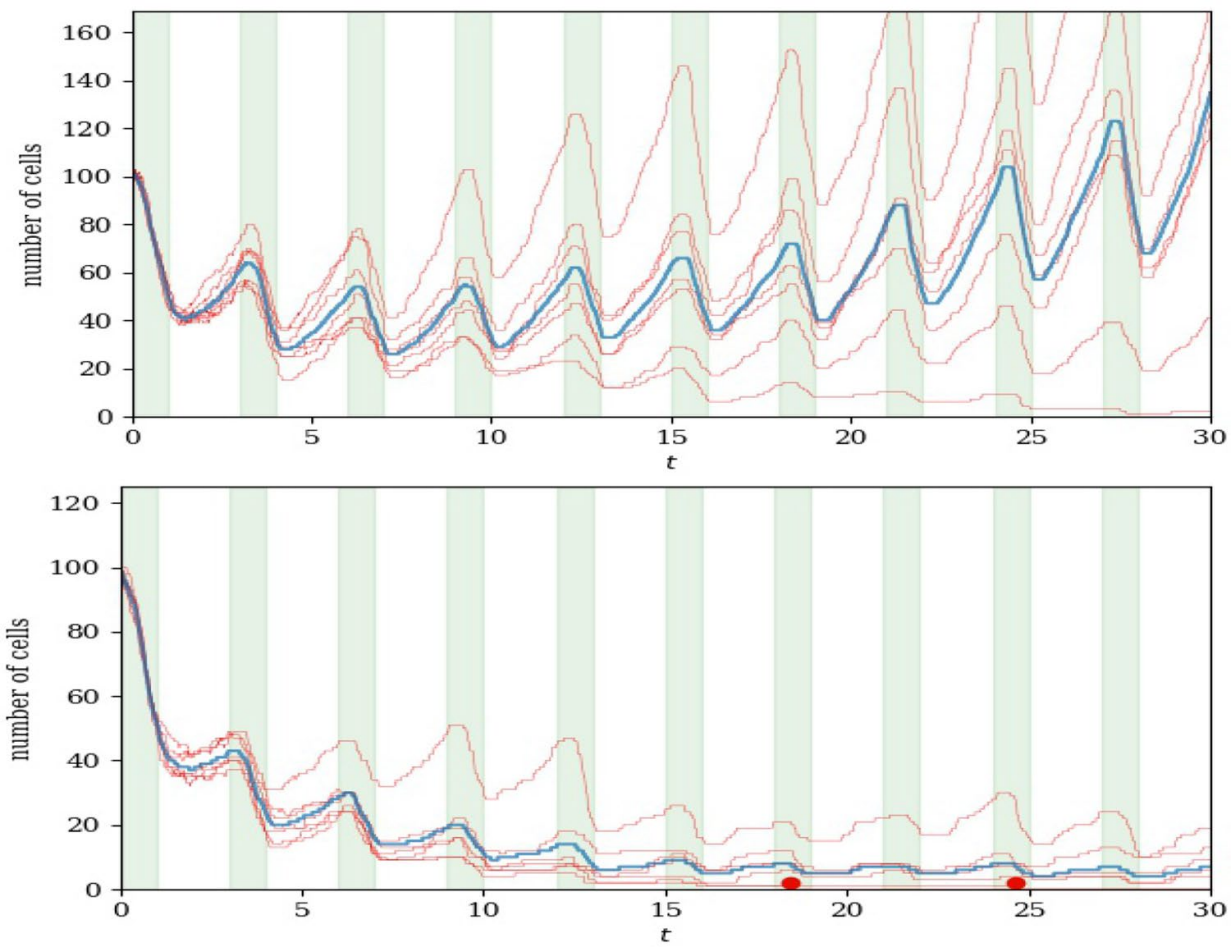
Number of cells as a function of time under multiple doses and recovery periods. Red lines are individual realisations and the ensemble average is shown in blue. Time intervals when the drug is applied are shaded green. Red dots indicate extinction times (the moment when the last cell dies, in one realisation). $\mu =1$ and $\delta =2.5$, $T=3$ and $T_d=1$. Top: $\lambda =0.4$. Bottom: $\lambda =0.25$. The initial population of 100 cells has regulator values chosen uniformly in [0, 1] (Color figure online)

Consider the effect of multiple doses on the size of the cell population. Recall that cells with $k_i(t)>0.5$ divide with rate $\lambda$ and cells with $k_i(t)<0.25$ die with rate $\mu$. The general decrease of regulator values during dosing periods pushes more cells both out of the division pool and into the death pool; the general increase in regulator values in recovery periods has the opposite effect. In the examples shown in Fig. 10, $T=3T_d$.

If the drug-induced death rate $\mu$ is sufficiently large, the typical increase in cell numbers during recovery periods is not sufficient to make up for the loss of cells during the preceding doses. Then, all cells will eventually be killed. However, the number of doses for complete extinction varies from realisation to realisation. In the lower part of Fig. 10, extinction of the cell population (indicated by a red dot) occurs before the end of the tenth dose in two of the realisations shown. On the other hand, if $\mu$ is sufficiently small, the population of cells will increase in the long run because more cells divide than are killed. This is the situation illustrated in the upper part of Fig. 10. In this parameter range, we observe observe a temporary decrease of average regulator values, and even observe extinction of the population in some realisations.

Can we calculate the threshold value of $\mu$ that defines ultimate extinction? We begin by noting that an individual cell’s fate depends on its initial regulator value. Firstly, cells with higher $k_i(0)$ spend less time in the death pool. Secondly, cells ‘remember’ their initial regulator value in the sense that $k_i(t)$ relaxes towards $k_i(0)$ in each recovery phase. An example of this is seen in Fig. 8 where, after three cycles of dose and recovery, all surviving cells are descended from the initial cell with highest regulator value. We provide an estimate of the threshold in the Supplementary Material.

The distribution of regulator values in the population of cells before the first dose is uniform in (0, 1). Each cycle of dose and recovery favours cells with larger regulator values (that spend less time in the death pool and more time in the division pool). This selection effect is an adaptation of the population akin to the development of drug resistance [25, 26] (even though it remains true that the drug, given enough time, kills all cells). Indeed, we may observe in the upper panel of Fig. 10 that the first few drug doses do reduce the cell population significantly, but the surviving cell population is able to recover.

## Discussion

In this work, we began by analysing the fate of a single cell, and a heterogeneous population of cells, under a model of sustained drug dose. Heterogeneity originates in the initial conditions: each cell’s starting *k* value is chosen randomly between 0 and 1. The effect of the drug, during a dose or doses, is to decrease each cell’s *k* value with timescale $1/\delta$. The cell population changes in size and distribution; those cells with $k<0.25$ are in the death pool, while those with $k>0.5$ are in the division pool. The survival probability of a typical cell, and survival or extinction of the whole population, is calculated. The mean time to extinction depends on the logarithm of the initial number of cells; the distribution of extinction times has a characteristic Gumbel limiting form. We continue by simulating a multiple-dose treatment where the cells are allowed to recover and divide between cycles. The balance between cell death and cell division determines the ultimate fate of the population after repeated rounds of drug dose and recovery.

The timescale $1/\delta$ characterises the potency of the drug (activity of the targeted protein). For example, in cancer cell cultures grown with and without drug treatment, and immunoblots of those cultures were created incubated with antibodies to activated phosphorylated ERK1 and ERK2 and total ERK1 and ERK2 [27]. The death and division rate is obtainable from tumour cell cultures under constant drug treatment in different concentrations. Yang et al  [19] used time-resolved microscopy to track the temporal change of the number of live and dead tumour cells in vitro. In xenograph models, tumour cells are injected subcutaneously or in the same organ as the tumour’s origin. The change in tumour size is quantified by surgically removing the tumour for ex vivo weighing, in vivo tumour volume measurement using calipers, within internal organs by employing magnetic resonance imaging, computed tomography, or ultrasound [28]. Additionally, organoids can be employed to measure longitudinal changes in tumour size [28].

In our model, we analyse the time to complete elimination of a heterogeneous tumour cell population. The fact that the extinction time, a natural endpoint in a stochastic model, is not available in deterministic models, may be seen as part of the general pattern that stochastic models are most relevant in small populations [9] (for example, the small residual cancer cell population after effective immunotherapy, the small initial population early in infection, the small fraction of the cells surviving antibiotic treatment  [29]). Even genetically-identical cells in a uniform in vitro environment differ in their response to drugs due to dynamic randomness in gene expression levels and other biochemical phenomena [30]. Tolerance where a bacterial population survives transient antibiotic exposure or resistance to a drug can be analysed to find the best time or concentration for a treatment [31]. Heterogeneity in cancer-cell populations results in resistance via changes to the drug target or downstream signalling network [26]. For example, in post-myeloproliferative neoplasm secondary acute myeloid leukemia the mutation in JAK2 increases nuclear $\beta$ catenin levels and its co-localization with TBL1, promoting growth and survival [32]. Additionally, resistance via persisters (dynamic non-genetic heterogeneity of clonal cell populations which produces metastable phenotypic variants) can be analysed [25].

Deterministic models of the effect of a drug on tumour cells using ordinary differential equations [5, 6] often have the advantage of easy implementation and analysis, but they do not naturally capture stochasticity or heterogeneity. Advances in molecular biology and the development of therapies that target intracellular signalling pathways [33–35] make it ever more important to consider heterogeneity of target cells. Biological heterogeneity also manifests itself in variable susceptibility to antibiotic treatments [29, 36]. Agent-based models overcome many shortcomings of simpler models because cells and their interactions are governed by stochastic rules, but they often require high computational power and running times and have large parameter spaces [12]. Here, based on a published agent-based model [12] where a heterogeneous cancer cell population is treated with a MEK inhibitor, we use stochastic modelling and analysis as a bridge between different types of models. The dynamics of a stochastic model is represented by relatively simple mathematical expressions which are reminiscent of deterministic models.

## Supplementary Information

Below is the link to the electronic supplementary material.

Supplementary file 1 (pdf 223 KB)

## Data Availability

No datasets were generated or analysed during the current study.

## Footnotes

1 https://github.com/VanThuyTruong/stochastic_pharmacodynamic.

2

$$\begin{aligned} p&=\exp \left( -nA\textrm{e}^{-\mu t}\right) \\ 1/p&= \exp \left( nA\textrm{e}^{-\mu t}\right) \\ \log (1/p)&=nA\textrm{e}^{-\mu t}\\ \log \left( \log (1/p) \right)&=\log (nA)-\mu t\\ \mu t&= \mu t_\textrm{max}- \log (\log (1/p)). \end{aligned}$$
