# Supplementary material for: Stochastic pharmacodynamics of a heterogeneous tumour-cell population
Source: J Pharmacokinet Pharmacodyn. 2025 May 5;52(3):28. doi: 10.1007/s10928-025-09974-7 (PMC12053371; doi:10.1007/s10928-025-09974-7)
Supplement: Supplementary file 1 — (pdf 223 KB) [file 10928_2025_9974_MOESM1_ESM.pdf]

Stochastic pharmacodynamics of a heterogeneous tumour-cell population - Supplementary

Van Thuy Truong[1,2], Paolo Vicini[3], James Yates [4], Vincent Dubois[2] and Grant Lythe[1]

[1]School of Mathematics, University of Leeds, Leeds LS2 9JT, UK.
Clinical Pharmacology and Quantitative Pharmacology, AstraZeneca, Granta Park, Cambridge, CB21 6GH, UK.
[3] Confo Therapeutics, Technologiepark 94, 9052 Ghent (Zwijnaarde), Belgium.
[4] DMPK, IVIVT, RD Research, GSK , Gunnels Wood Road, Stevenage, Hertfordshire, SG1 2NY, United Kingdom.

9th March 2025

1 Non-constant death rate: single sustained dose

In this Section, the death rate function in Section 2 is not w_0 but w_1 where

$$w_1(k) = \max\{\mu(1-4k), 0\}. \tag{1}$$

Thus a cell's survival probability is a function of its initial regulator value, given by

$$\begin{aligned} s_1(t,k) &= \exp\left(-\mu \int_{t_k}^{t} (1-4k(s))\mathrm{d}s\right) \\ &= \exp\left(-\mu(t - t_k - \frac{4k}{\delta}(1+\mathrm{e}^{-\delta(t-t_k)}))\right). \end{aligned} \tag{2}$$

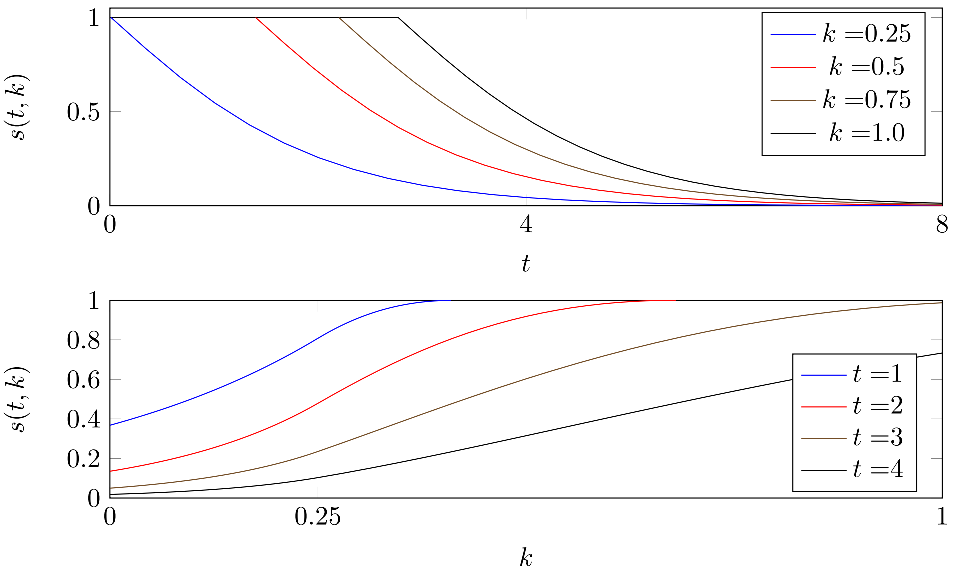

Figure 1: Upper: each line is fixed k and $s(t,k)$ is the probability that a cell, whose initial regulator value is k, is still alive at time t. Lower: each line is fixed t and shows the probability that a cell, whose initial regulator value is k, is still alive. The formula used is (2), with $\mu = 1$ and $\delta = 0.5$.

As $\delta t \to \infty$,

$$s_1(t,k) \to \begin{cases} e^{-\mu t} e^{4k\mu/\delta} & k \leq \frac{1}{4} \\ e^{-\mu t}(4k)^{\mu/\delta} e^{\mu/\delta} & k > \frac{1}{4}. \end{cases} \tag{3}$$

Thus

$$S_1(t) = \int_0^1 s_1(t,k)\mathrm{d}k \to A_1 e^{-\mu t} \qquad \text{as } \delta t \to \infty, \tag{4}$$

where

$$A_1 = e^{\mu/\delta} \frac{4^{\mu/\delta} - \frac{1}{4}}{1 + \frac{\mu}{\delta}} + \frac{\delta}{4\mu}(e^{\mu/\delta} - 1). \tag{5}$$

2 Estimate of critical value - multiple doses with recovery

Given λ, T and T_d, what is the minimum value of μ necessary to guarantee eventual extinction? Figure 2 summarises numerical results at different values of μ. Blue dots represent the mean number of cells still alive after 100 cycles of dose and recovery. If μ is sufficently large then the mean number of cells is small (and all cells are eliminated in some realisations). At smaller values of μ, the population grows in the long run. In a deterministic model, there is a sharp threshold between behaviours in the late-time limit. In a stochastic model, however, the probability of extinction is non-zero even when μ is below the threshold. In both cases, the greater the number of doses, the sharper the transition.

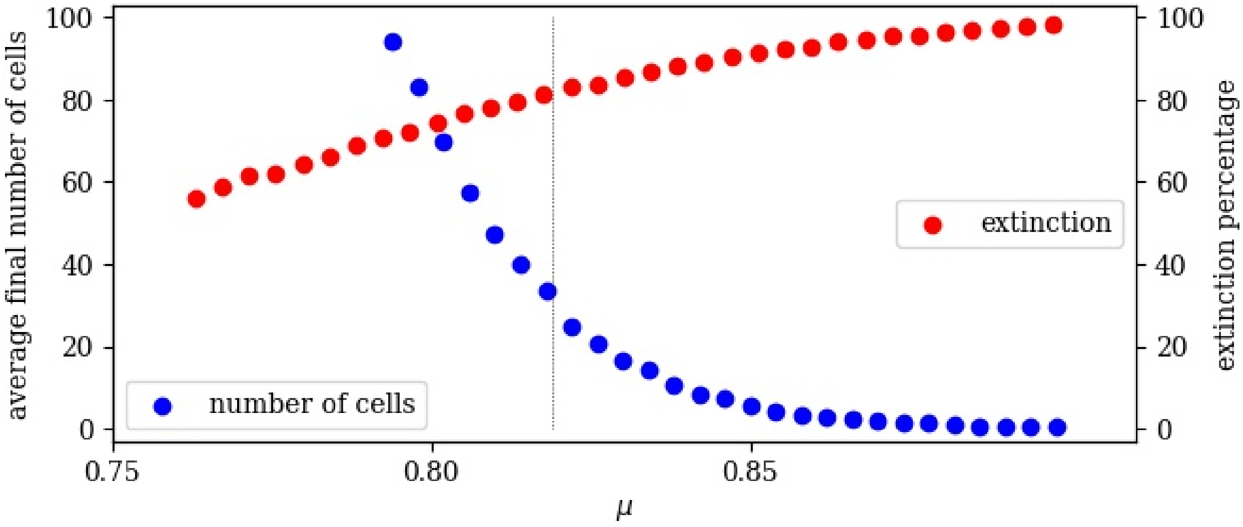

Figure 2: Each blue dot represents the mean number of cells still alive after 100 doses; Each red dot represents the extinction percentage calculated from 10000 realisations. The initial number of cells is 100 and the parameter values are $\lambda = 0.25$, $\alpha = 2$, $\delta = 2.5$, $T = 3$ and $T_d = 1$. The dotted vertical line is the estimated value of μ, below which ultimate extinction is certain given a sufficient number of doses, calculated using (6).

With these caveats, it is useful to constract an approximation for the critical value of μ and examine its parameter dependence. We may estimate the critical value μ_c from the relation imposing a balance between death anad division of tumpur cells:

$$\mu \times \text{mean time spent in death pool} \qquad = \qquad \lambda \times \text{mean time spent in division pool.}$$

Applying this relation exactly would require knowledge of the distribution of regulator values after many cycles of dose and recovery. We obtain an estimate of μ_c by considering the times T_1, T_2, T_3 and T_4 that characterise entry to and exit from the death and divison pools, as shown in Figure 3. Using the approximations given in Figure 3, we estimate that the critical value, μ_c, satisfies the condition

$$\mu_c(T_3 - T_2) = \lambda(T_1 + T - T_4) \tag{6}$$

We note, firstly, that μ_c is proportional to λ, the division rate of cells when they are in the division pool. Next, we observe that μ_c is a decreasing function of δ and of T_d/T. That is, faster-acting drugs, and drugs that are applied for a longer fraction of the total cycle time, kill cancer cells more rapidly. The dependence on the parameter α that describes the rate of relaxation of regulator values in the recovery periods, is more complicated because speed of recovery affects times spent in both death and division pools.

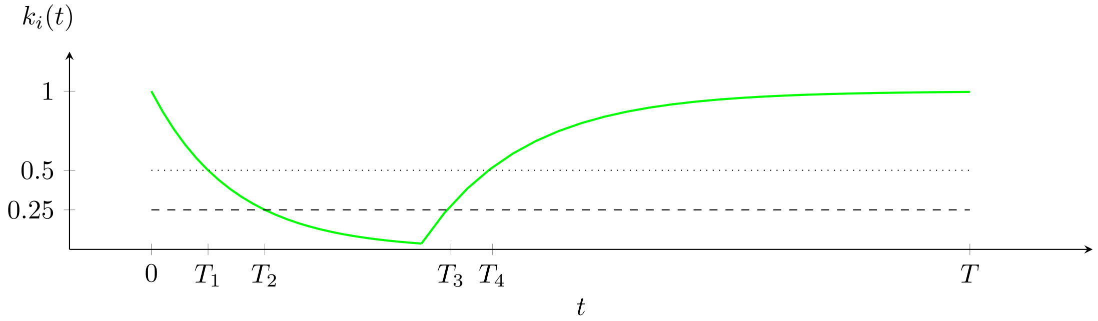

Figure 3: Constructing the approximation. The green line is the regulator value of one cell through one round of dose and recovery. Dotted line: $k = 0.5$. Dashed line: $k = 0.25$. We use the following approximations: $T_1 = \frac{1}{\delta}\log 2$, $T_2 = \frac{1}{\delta}\log 4$, $T_3 = T_d + \frac{1}{\alpha}\log\frac{4}{3}$, $T_4 = T_d + \frac{1}{\alpha}\log 2$.
